# The Role of Bacteria in the Pathogenesis of Ulcerative Colitis

**DOI:** 10.1155/2012/704953

**Published:** 2012-04-24

**Authors:** Maiko Sasaki, Jan-Michael A. Klapproth

**Affiliations:** Division of Digestive Diseases, Atlanta Veterans Administration Medical Center, Emory University, Whitehead Biomedical Research Building, Suite 201, 615 Michael Street, Atlanta, GA 30322, USA

## Abstract

Factors implicated in the pathophysiology of ulcerative colitis (UC) are an abnormal immune response, defect in intestinal epithelial barrier function, and gut microbiota. Currently, it is unclear whether specific bacterial strains are responsible for the induction of intestinal inflammation, but increased bacterial tissue invasion has been described in affected UC patients. Further, a quantitative and qualitative microbial imbalance in UC, defined as dysbiosis, has been characterized by an increase in *Rhodococcus spp.*, *Shigella spp.*, and *Escherichia spp.*, but a decrease in certain *Bacteroides spp.*. More specifically, *Campylobacter spp.*, *Enterobacteriae*, and enterohepatic *Helicobacter* were more prevalent in tissue sample from UC patients subjected to molecular detection methods, but not controls. In addition, serologic testing identified *Fusobacterim varium* as a potential contributor to the intestinal inflammation in UC. Interestingly, in-situ hybridization studies have shown anti-inflammatory *Lactobacillus spp.* and *Pediococcus spp.* were absent in samples from subjects affected by UC. Therefore, dysbiosis is a factor in the pathogenesis of UC.

## 1. Introduction

The gut microbiota consists predominantly of phyla members *Bacteroidetes* and *Firmicutes*, and to a lesser extent of *Actinobacteria* and *Proteobacteria *[1, 2]. There is an estimated 500 to 1,000 different bacterial species represented throughout the human intestine [3]. The number of colony forming units has been calculated at a range from 10^13^ to 10^14^, exceeding the number of human cells by factor of 10 [4]. The enteric bacterial flora as a whole is essential to the normal development and function of the intestine. Salvage of unabsorbed carbohydrates, converted into short-chain fatty acids by bacterial enzymes, is an essential energy source for intestinal epithelial cell and barrier function. The colonic microflora is also central to the synthesis of vitamins B and K [5], and maintenance of intestinal innate and adaptive immune response [3]. On the other side, there is mounting evidence that the intestinal microflora can induce, transfer, and prevent conditions like obesity, type I diabetes, and inflammatory bowel disease (IBD) with a detrimental effect on human health [6]. The focus of this paper is to summarize the evidence for a role of enteric bacteria in the pathogenesis of ulcerative colitis (UC).

Computational data mining by canonical correlation analysis confirmed the critical and disease-relevant interaction of mucosa-associated bacteria and host in IBD [7]. Bacterial interactions with the host were found to be of cyclic nature with an increase in disease inducing bacterial strains and host immune response during active intestinal inflammation. But dysbiosis, defined as quantitative and qualitative microbial imbalance in the gut, is only one factor contributing to intestinal inflammation as seen in UC. Investigating the mucosal immune response, it has been shown that patients with UC mount an immunoglobulin response against endogenous bacterial components. In UC, the DNase-sensitive neutrophil autoantibody with atypical perinuclear distribution (pANCA) was found to be directed against two bacterial antigens: an unidentified 100 kDa protein from *Bacteroides caccae* and outer membrane porin C (OmpC) from *E. coli* strains [8]. Given pANCA's low sensitivity, it should not be used as a screening tool for IBD in the general population, but might aid in distinguishing UC from Crohn's disease (CD) when used in combination with anti-*Saccharomyces cerevisiae* antibody (ASCA), in particular when surgery is entertained. A combination of positive pANCA and negative ASCA predicted UC correctly in 64% of cases [9]. Other more controversial findings regarding the role of pANCA in UC include its association with severe, relapsing and therapy-refractory left-sided disease, early colectomy for an aggressive course, and higher requirements for immunomodulatory therapy [9]. Additional immunological findings revealed that the intestinal mucosa of patients suffering from UC is infiltrated with Th17 cells [10], stimulated by IL-23, a cytokine released by antigen-presenting cells in response to bacterial stimulation [11]. Also, genomewide association studies [12] have detected additional critical factors for the pathogenesis of UC. These include hepatocyte nuclear factor 4 (HNF4A), a protein regulating intercellular cell junctions, like desmosomes, tight and adherence junctions [13], and laminin *β*1 subunit (LAMB1), anchoring epithelium to the underlying basement membrane. Interestingly, laminin has been shown previously to be absent from the surrounding membranes in inflamed tissue section affected by UC [14]. Other genes identified in the association studies were E-cadherin (CDH1), a protein member of adherens junctions and transcription factor guanine nucleotide binding protein alpha 12 (GNA12). Common to HNF4A, LAMB1, CDH1, and GNA12 is the fact that these genes are all involved in the maintenance of intestinal epithelial cell integrity and barrier function [15].

The findings outlined above have defined the currently accepted hypothesis for the development of IBD, “Pathogenic intestinal bacteria and/or infectious agents initiate and perpetuate the inflammation of the gut through disruption of tolerance towards the commensal microbiota in an individual with genetic vulnerability.” [16]. 

## 2. Dysbiosis in Ulcerative Colitis

Currently, it is not clear which factors initiate or maintain the inflammatory process in UC. There are opposing views whether an imbalance in gut flora even occurs in UC [17], but the evidence presented here does suggest that this is the case. Large epidemiological studies have addressed the question whether a trigger event lead to dysbiosis in UC. In a study from Spain with an average follow-up time of 3.5 years, the estimated incidence of developing both CD and UC was significantly elevated in patients with an identifiable episode of acute gastroenteritis [18]. For the control cohort without an episode of gastroenteritis, the incidence of IBD was calculated at 29.7/100,000 person years, but it increased to 68.4/100,000 person years for patients with previously identified episode of bacterial intestinal infection. In this study, the most commonly identified bacterial pathogen as a cause of enteric infection was *Campylobacter spp.*, followed by *Salmonella spp.* and *Shigella spp.*. Similarly, a gender and age-matched study from Denmark identified an increased risk for the development of CD and UC after infection with *Campylobacter spp.* or *Salmonella spp. *[19, 20]. The risk for CD and UC was highest during the first year following infection, in particular for inpatients, and remained elevated during the ensuing 15 years. These findings were disputed in another study from Denmark, which determined the incidence rate ratio of populations with or without exposure to *Campylobacter spp.* or *Salmonella spp. *[21]. Contrary to previously published results, the risk of developing CD and UC was found to be independent of positive or negative stool studies. The authors concluded that the increased discovery of previous *Campylobacter spp.* and *Salmonella spp.* infection at the time of diagnosis of CD or UC was due to increased rates of stool testing, consistent with detection bias.

However, it is still attractive to speculate that an acute enteric infection leads to possibly chronic changes in intestinal milieu and/or enteric microflora, or both. Indeed, there are a number of excellent studies that have investigated the quantitative and qualitative changes in the composition of the enteric flora in UC. Attempting to enumerate the number of bacteria in IBD patients, tissue samples were subjected to either enumeration by culture or quantitative rRNA hybridization [22]. Samples from both CD and UC subjects contained significantly more bacteria when compared to normal control tissue, and a gradual increase was observed from noninflamed to inflamed biopsy material [23]. In these experiments, imaging identified bacteria localized within the mucus layer without directly adhering to the underlying lamina propria. Additional results from another laboratory also showed increased bacterial adherence and invasion of epithelial cells and an enhanced inflammatory response [24]. Similarly, when determined by real-time quantitative PCR, biopsy samples from individuals with newly diagnosed UC harbored a significantly higher number of mucosa-associated bacteria in comparison to samples obtained from CD or healthy controls [25]. Similarly, 16S rRNA-based amplification revealed increased total CFU for aerobes, facultative anaerobes, and Gram negative bacteria in a pediatric population [26]. At the same time, a decreased number of *Bacteroides vulgatus* were amplified in comparison to healthy control subjects. In this study, the only Gram negative bacterial species identified in pediatric UC was *Escherichia coli*. In an opposing view, qualitative analysis revealed a similar distribution of unclassified *Bacteroidetes* in UC and healthy control samples [27]. Distinguishing between the microbiota of inflamed and noninflamed samples, it appears that with the onset of inflammation, bacterial diversity declines. These findings have been supported by other qualitative studies investigating the enteric flora of patients with UC. Denaturing gradient gel electrophoresis with universal and *Bacteroidetes*-specific primers and multivariant analysis revealed reduced diversity of predominant bacteria commonly found in healthy volunteers [28]. Species conspicuously absent from the enteric flora in 13 patients with documented UC included *Bacteroides vulgatus*, *B. ovatus*, *B. uniformis*, and *Parabacteroides spp.*. Similarly, in a landmark study by Frank et al., abnormal gut flora was identified in patients with CD and UC [29]. When subjected to culture-independent rRNA sequence analysis, common to both diseases was a reduction of phyla *Bacteroidetes* and *Firmicutes*. Both phyla promote gut health through the production of short-chain fatty acids, which are the primary energy source for intestinal epithelial cells, critical for the maintenance of barrier integrity [30, 31] and suppression of immune activation [32]. Depletion of short-chain fatty acid-producing organisms possibly deprives already vulnerable intestinal epithelial cells, leading to invasion of commensal or low-pathogenic bacteria with subsequent activation of immunocompetent cells.

But dysbiosis, as seen in UC, includes additional pathophysiological changes relevant to intestinal inflammation. Fluorescence in situ hybridization detected invasive bacteria in 83% of tissue samples from patients with UC as opposed to none in negative controls [33]. The organisms invading terminal ileum and colon of UC affected individuals were identified as *Proteobacteria*, *Clostridium*, *Enterobacteriae*, *Bacteroides*, and *Prevotella*. These investigations have opened additional trials attempting to identify a single or multiple disease-specific bacterial strains. A recently published study of twins affected and not affected by IBD-identified potentially pathogenic bacteria that were more frequently identified in patients suffering from UC [34]. These strains included *Rhodococcus spp.*, *Shigella spp.*, *Escherichia spp.*, and *Stenotrophomonas spp.*. At the same time, bacteria with anti-inflammatory properties were more frequently identified in siblings not affected by UC, including *Faecalibacterium prausnitzii*. A reduction in *F. prausnitzii* was recently shown to be associated with a higher risk of postoperative recurrence of CD, as documented by endoscopy at six months [35]. The proposed anti-inflammatory effect of *F. prausnitzii* was attributed to the attenuated activation of NF-**κ**B and MAP3K8, with subsequent reduction of IL-8 expression.

## 3. Specific Bacteria Increased in UC

Comparing gut tissue samples obtained from patients with both infectious diarrhea and UC microscopically, both diseases show a significant overlap in pathological findings [36]. PCR and sequencing analyses identified *Campylobacter spp.* in 74% of biopsy samples in a cohort of 69 patients with confirmed UC as opposed to 23% from healthy controls, even without the history of acute gastroenteritis [37]. Specifically, nested PCR for *Campylobacter concisus* was positive, and it was more common in UC samples when compared to healthy controls; 33% versus 11%, respectively. In addition, *Campylobacter ureolyticus* was positive in 22% of UC biopsy material compared to 3% of control samples, with supporting evidence in a similar study from India [38]. These findings led the authors to speculate that a specific immunological defect in UC results in the inability to eliminate *Campylobacter spp.*. Independent of the underlying host defect, a possible mechanistic explanation for a role of *Campylobacter spp.* in the pathogenesis of UC has recently been provided. *Campylobacter jejuni* was found to facilitate internalization and translocation of commensal, noninvasive *E. coli* strains via the transcellular and paracellular pathways in vitro and in vivo [39, 40]. These findings might indicate that in UC, *Campylobacter spp.* induce an inflammatory cascade that starts with an episode of acute gastroenteritis.

Besides *Campylobacter spp.*, ribosomal intergenic spacer analysis with subsequent sequencing analysis of unique PCR bands detected 3 to 4 logs higher abundance of *Enterobacteriae* belonging to the B2 and D phylogenetic groups in both CD and UC [41]. This might be in support of an argument for the role of specific bacteria in UC, as pathogenic *E. coli* strains belong predominantly to group B2 and to a lesser degree to group D. In a related study, *E. coli* isolates from patients with CD and UC displayed higher-adhesion indices in comparison to strains from normal controls [42]. *E. coli* associated with UC tissue harbor more adhesion/virulence determinants than strains from CD biopsy samples. More specifically, *E. coli* strains positive for pathogenicity factors *ompA*, *afae*, and *USP* were more likely to be identified in patients suffering from UC [43]. As mentioned before, these *E. coli* belonged to phylotype B2 and D, and were associated with active inflammation. This particular study also described an increased intracellular survival of invading *E. coli* in macrophage cultures in vitro, consistent with increased pathogenicity of UC bacterial isolates.

Adding to the list of potentially pathogenic bacteria in UC, PCR-based methods detected increased enterohepatic *Helicobacter* in subjects with CD and UC in comparison to members of the control population [44, 45]. In contrast to subjects suffering from IBD, the control population had a higher likelihood of infection with *Helicobacter pylori*. Given these results, the authors speculated that cytolethal toxin from enterohepatic *Helicobacter *plays a potential role in the intestinal inflammation of IBD. However, the molecular detection methods identifying *Campylobacter spp.*, *E. coli*, and enterohepatic *Helicobacter* in patients with UC await confirmation by an alternative method.

Alternatively, serological markers have been used to implicate specific bacterial strains in the pathogenesis of UC. *Bacteroides ovatus* caused an increased IgG and IgA antibody response in patients with IBD as opposed to normal controls [46]. This study identified and implicated a novel 19.5 kDa prominent antigen in the pathogenesis of both CD and UC. Similarly, UC patients were more likely to be seropositive for antibodies directed against *Fusobacterium varium* antigens (40%) in comparison to normal controls (16%) [47]. Correlating disease activity with seropositivity, patients with elevated *F. varium* immunoglobulins were more likely to be symptomatic and harbor extensive disease. In vitro investigations of *F. varium* by the same group have shown that this particular commensal strain invades epithelial cell lines, and induces expression of proinflammatory cytokine mRNAs, including IL-8, TNF-*α*, MCP-1, and IL-6 [48]. Further, in vivo experiments identified *F. varium* to produce very high concentrations of butyric acid, causing intestinal lesions in mice, similar to those observed in human UC [49]. In turn, elevated butyric acid was shown to increase the activity of proapoptotic pathways with subsequent erosions, a possible pathophysiological mechanism in UC [50]. These findings have led to three clinical trials investigating the efficacy of antibiotics in UC to specifically suppress *F. varium *[51–53]. Consistently, antimicrobial therapy resulted in significantly decreased CFUs and antibody titers directed against *F. varium*, improved endoscopic and histological scores, and clinical response at 12 months after treatment. In addition, patients treated with a combination of amoxicillin, tetracycline, and metronidazole for 14 days were more likely to discontinue steroid therapy at 3, 6, and 12 months.

## 4. Specific Bacteria Decreased in UC

It is becoming increasingly clear that the quality and quantity of intestinal microflora vary with disease activity, and active inflammation is not solely due to an increase of specific bacterial strains. During active UC, anti-inflammatory *Lactobacillus salivarius*, *L. manihotivorans*, and *Pediococcus acidilactici* were absent in fecal samples analyzed by fluorescence in situ hybridization [54]. With UC in remission, these strains reappeared. In a related study, *Bifidobacterium spp.* were identified in decreased numbers in both inflamed UC and CD while *Lactobacillus spp.* was unchanged during active UC [55]. The same study commented on the reduced thickness of the mucus layer when compared to controls.

## 5. Summary and Conclusion

Here we have discussed the roles of enteric bacteria in UC. The bacterial strains that have been associated with various roles are summarized in Table 1. It is conceivable that pathogenic bacteria, including *Campylobacter spp.*, *Salmonella spp.,* and other currently unidentified pathogens, take the lead in initiating the inflammatory process in UC with an episode of acute gastroenteritis. Pathogenic and commensal strains and their effector proteins weaken the intestinal lining through production of high concentrations of butyric acid and translocation of nonpathogenic bacteria in genetically susceptible patients with a defect in the intestinal epithelial barrier function. Extensive immune activation due to breakdown of the intestinal barrier provides bacteria access to the gut mucosal immune system, resulting in uncontrolled inflammation and dysbiosis.

## Figures and Tables

**Table 1:** Summary of enteric bacteria and their contribution to intestinal inflammation in UC.

Role in UC	Strain	Location	Reference
Initiation of inflammation	*Campylobacter spp.*	Europe	[19, 20]
	*Salmonella spp.*	Europe	[19, 20]
	*Shigella spp.*	Europe	[19, 20, 34]

Proinflammatory	*Campylobacter spp.*	Europe	[37]
	*Escherichia coli*	Europe and America	[26, 34, 39–43]
	*Rhodococcus spp.*	Europe	[34]
	*Stenotrophomonas spp.*	Europe	[34]
	Enterohepatic *Helicobacter *	Europe and America	[44, 45]
	*Bacteroides ovatus*	Asia	[46]
	*Fusobacterium varium*	Asia	[47]

Anti-inflammatory	*Bacteroides spp.*	Europe and America	[26, 28, 29]
	*Firmicutes*	America	[29]
	*Faecalibacterium prausnitzii*	Europe	[34]
	*Lactobacillus spp.*	Europe	[54]
	*Pediococcus acidilactici *	Europe	[54]

## References

[1] Zoetendal EG, Rajilić-Stojanović M, De Vos WM (2008). High-throughput diversity and functionality analysis of the gastrointestinal tract microbiota. *Gut*.

[2] Tannock GW (2010). The bowel microbiota and inflammatory bowel diseases. *International Journal of Inflammation*.

[3] Sears CL (2005). A dynamic partnership: celebrating our gut flora. *Anaerobe*.

[4] Hooper LV, Gordon JI (2001). Commensal host-bacterial relationships in the gut. *Science*.

[5] Cummings JH, Macfarlane GT (1997). Collaborative JPEN-Clinical Nutrition Scientific Publications role of intestinal bacteria in nutrient metabolism. *Journal of Parenteral and Enteral Nutrition*.

[6] Serino M, Luche E, Chabo C, Amar J, Burcelin R (2009). Intestinal microflora and metabolic diseases. *Diabetes and Metabolism*.

[7] Presley LL, Ye J, Li X (2012). Host-microbe relationships in inflammatory bowel disease detected by bacterial and metaproteomic analysis of the mucosal-luminal interface. *Inflammatory Bowel Diseases*.

[8] Cohavy O, Bruckner D, Gordon LK (2000). Colonic bacteria express an ulcerative colitis pANCA-related protein epitope. *Infection and Immunity*.

[9] Prideaux L, De Cruz P, Ng SC Serological antibodies in inflammatory bowel disease: a systematic review.

[10] McGovern D, Powrie F (2007). The IL23 axis plays a key role in the pathogenesis of IBD. *Gut*.

[11] Sarra M, Pallone F, MacDonald TT, Monteleone G (2010). IL-23/IL-17 axis in IBD. *Inflammatory Bowel Diseases*.

[12] Barrett JC, Lee JC, Lees CW (2009). Genome-wide association study of ulcerative colitis identifies three new susceptibility loci, including the HNF4A region. *Nature Genetics*.

[13] Battle MA, Konopka G, Parviz F (2006). Hepatocyte nuclear factor 4*α* orchestrates expression of cell adhesion proteins during the epithelial transformation of the developing liver. *Proceedings of the National Academy of Sciences of the United States of America*.

[14] Schmehl K, Florian S, Jacobasch G, Salomon A, Korber J (2000). Deficiency of epithelial basement membrane laminin in ulcerative colitis affected human colonic mucosa. *International Journal of Colorectal Disease*.

[15] Lees CW, Barrett JC, Parkes M, Satsangi J (2011). New IBD genetics: common pathways with other diseases. *Gut*.

[16] Matricon J, Barnich N, Ardid D (2010). Immunopathogenesis of inflammatory bowel disease. *Self/Nonself. Immune Recognition and Signaling*.

[17] Gophna U, Sommerfeld K, Gophna S, Doolittle WF, Veldhuyzen Van Zanten SJO (2006). Differences between tissue-associated intestinal microfloras of patients with Crohn’s disease and ulcerative colitis. *Journal of Clinical Microbiology*.

[18] Rodríguez LAG, Ruigómez A, Panés J (2006). Acute gastroenteritis is followed by an increased risk of inflammatory bowel disease. *Gastroenterology*.

[19] Gradel KO, Nielsen HL, Schønheyder HC, Ejlertsen T, Kristensen B, Nielsen H (2009). Increased short- and long-term risk of inflammatory bowel disease after salmonella or campylobacter gastroenteritis. *Gastroenterology*.

[20] Ternhag A, Törner A, Svensson A, Ekdahl K, Giesecke J (2008). Short- and long-term effects of bacterial gastrointestinal infections. *Emerging Infectious Diseases*.

[21] Jess T, Simonsen J, Nielsen NM (2011). Enteric salmonella or campylobacter infections and the risk of inflammatory bowel disease. *Gut*.

[22] Schultsz C, Van den Berg FM, Kate FWT, Tytgat GNJ, Dankert J (1999). The intestinal mucus layer from patients with inflammatory bowel disease harbors high numbers of bacteria compared with controls. *Gastroenterology*.

[23] Swidsinski A, Ladhoff A, Pernthaler A (2002). Mucosal flora in inflammatory bowel disease. *Gastroenterology*.

[24] Swidsinski A, Loening-Baucke V, Herber A (2009). Mucosal flora in Crohn's disease and ulcerative colitis—an overview. *Journal of Physiology and Pharmacology*.

[25] Bibiloni R, Mangold M, Madsen KL, Fedorak RN, Tannock GW (2006). The bacteriology of biopsies differs between newly diagnosed, untreated, Crohn’s disease and ulcerative colitis patients. *Journal of Medical Microbiology*.

[26] Conte MP, Schippa S, Zamboni I (2006). Gut-associated bacterial microbiota in paediatric patients with inflammatory bowel disease. *Gut*.

[27] Sepehri S, Kotlowski R, Bernstein CN, Krause DO (2007). Microbial diversity of inflamed and noninflamed gut biopsy tissues in inflammatory bowel disease. *Inflammatory Bowel Diseases*.

[28] Noor SO, Ridgway K, Scovell L (2010). Ulcerative colitis and irritable bowel patients exhibit distinct abnormalities of the gut microbiota. *BMC Gastroenterology*.

[29] Frank DN, Amand ALS, Feldman RA, Boedeker EC, Harpaz N, Pace NR (2007). Molecular-phylogenetic characterization of microbial community imbalances in human inflammatory bowel diseases. *Proceedings of the National Academy of Sciences of the United States of America*.

[30] Galvez J, Rodríguez-Cabezas ME, Zarzuelo A (2005). Effects of dietary fiber on inflammatory bowel disease. *Molecular Nutrition and Food Research*.

[31] Lara-Villoslada F, De Haro O, Camuesco D (2006). Short-chain fructooligosaccharides, in spite of being fermented in the upper part of the large intestine, have anti-inflammatory activity in the TNBS model of colitis. *European Journal of Nutrition*.

[32] Segain JP, Raingeard De La Blétière D, Bourreille A (2000). Butyrate inhibits inflammatory responses through NF*κ*B inhibition: implications for Crohn’s disease. *Gut*.

[33] Kleessen B, Kroesen AJ, Buhr HJ, Blaut M (2002). Mucosal and invading bacteria in patients with inflammatory bowel disease compared with controls. *Scandinavian Journal of Gastroenterology*.

[34] Lepage P, Hösler R, Spehlmann ME (2011). Twin study indicates loss of interaction between microbiota and mucosa of patients with ulcerative colitis. *Gastroenterology*.

[35] Sokol H, Pigneur B, Watterlot L (2008). Faecalibacterium prausnitzii is an anti-inflammatory commensal bacterium identified by gut microbiota analysis of Crohn disease patients. *Proceedings of the National Academy of Sciences of the United States of America*.

[36] Kumar NB, Nostrant TT, Appelman HD (1982). The histopathologic spectrum of acute self-limited colitis (acute infectious-type colitis). *American Journal of Surgical Pathology*.

[37] Mukhopadhya I, Thomson JM, Hansen R, Berry SH, El-Omar EM, Hold GL (2011). Detection of campylobacter concisus and other campylobacter species in colonic biopsies from adults with ulcerative colitis. *PLoS One*.

[38] Verma R, Verma AK, Ahuja V, Paul J (2010). Real-time analysis of mucosal flora in patients with inflammatory bowel disease in India. *Journal of Clinical Microbiology*.

[39] Kalischuk LD, Inglis GD, Buret AG (2009). Campylobacter jejuni induces transcellular translocation of commensal bacteria via lipid rafts. *Gut Pathogens*.

[40] Lamb-Rosteski JM, Kalischuk LD, Inglis GD, Buret AG (2008). Epidermal growth factor inhibits Campylobacter jejuni-induced claudin-4 disruption, loss of epithelial barrier function, and Escherichia coli translocation. *Infection and Immunity*.

[41] Kotlowski R, Bernstein CN, Sepehri S, Krause DO (2007). High prevalence of Escherichia coli belonging to the B2+D phylogenetic group in inflammatory bowel disease. *Gut*.

[42] Schippa S, Conte MP, Borrelli O (2009). Dominant genotypes in mucosa-associated *Escherichia coli* strains from pediatric patients with inflammatory bowel disease. *Inflammatory Bowel Diseases*.

[43] Sepehri S, Khafipour E, Bernstein CN (2011). Characterization of *Escherichia coli* isolated from gut biopsies of newly diagnosed patients with inflammatory bowel disease. *Inflammatory Bowel Diseases*.

[44] Bohr URM, Glasbrenner B, Primus A, Zagoura A, Wex T, Malfertheiner P (2004). Identification of enterohepatic Helicobacter species in patients suffering from inflammatory bowel disease. *Journal of Clinical Microbiology*.

[45] Thomson JM, Hansen R, Berry SH (2011). Enterohepatic helicobacter in ulcerative colitis: potential pathogenic entities?. *PLoS One*.

[46] Saitoh S, Noda S, Aiba Y (2002). Bacteroides ovatus as the predominant commensal intestinal microbe causing a systemic antibody response in inflammatory bowel disease. *Clinical and Diagnostic Laboratory Immunology*.

[47] Minami M, Ando T, Okamoto A (2009). Seroprevalence of Fusobacterium varium in ulcerative colitis patients in Japan. *FEMS Immunology and Medical Microbiology*.

[48] Ohkusa T, Yoshida T, Sato N, Watanabe S, Tajiri H, Okayasu I (2009). Commensal bacteria can enter colonic epithelial cells and induce proinflammatory cytokine secretion: a possible pathogenic mechanism of ulcerative colitis. *Journal of Medical Microbiology*.

[49] Ohkusa T, Okayasu I, Ogihara T, Morita K, Ogawa M, Sato N (2003). Induction of experimental ulcerative colitis by Fusobacterium varium isolated from colonic mucosa of patients with ulcerative colitis. *Gut*.

[50] Yoshida T, Sekine T, Aisaki KI, Mikami T, Kanno J, Okayasu I (2011). CITED2 is activated in ulcerative colitis and induces p53-dependent apoptosis in response to butyric acid. *Journal of Gastroenterology*.

[51] Ohkusa T, Kato K, Terao S (2010). Newly developed antibiotic combination therapy for ulcerative colitis: a double-blind placebo-controlled multicenter trial. *American Journal of Gastroenterology*.

[52] Nomura T, Ohkusa T, Okayasu I (2005). Mucosa-associated bacteria in ulcerative colitis before and after antibiotic combination therapy. *Alimentary Pharmacology and Therapeutics*.

[53] Ohkusa T, Nomura T, Terai T (2005). Effectiveness of antibiotic combination therapy in patients with active ulcerative colitis: a randomized, controlled pilot trial with long-term follow-up. *Scandinavian Journal of Gastroenterology*.

[54] Bullock NR, Booth JCL, Gibson GR (2004). Comparative composition of bacteria in the human intestinal microflora during remission and active ulcerative colitis. *Current Issues in Intestinal Microbiology*.

[55] Fyderek K, Strus M, Kowalska-Duplaga K (2009). Mucosal bacterial microflora and mucus layer thickness in adolescents with inflammatory bowel disease. *World Journal of Gastroenterology*.

